# Single-Bolus Tinzaparin Anticoagulation in Extended Hemodialysis Session: A Feasibility Study

**DOI:** 10.34067/KID.0000000000000098

**Published:** 2023-03-15

**Authors:** Benoît Harvey, Jean-Philippe Lafrance, Naoual Elftouh, Michel Vallée, Louis-Philippe Laurin, Annie-Claire Nadeau-Fredette

**Affiliations:** 1 Division of Nephrology, Maisonneuve-Rosemont Hospital, Montreal, Quebec, Canada; 2 Maisonneuve-Rosemont Hospital Research Center, Montreal, Quebec, Canada

**Keywords:** home hemodialysis, tinzaparin, anticoagulation, nocturnal hemodialysis, low-molecular weight heparin

## Abstract

**Key Points:**

- A single bolus of tinzaparin is effective for 8-hour hemodialysis session.
- Eight-hour simulation sessions with anti-Xa monitoring are useful to adjust tinzaparin dose.

**Background:**

Few studies have assessed the use of low-molecular weight heparins for anticoagulation during extended hemodialysis (HD) sessions. This study aimed to evaluate the efficacy of a single bolus of tinzaparin for anticoagulation of the extracorporeal circuit and dialyzer in 8-hour HD sessions.

**Methods:**

This single-center study included all patients who underwent a single 8-hour simulation session as part of their nocturnal home HD training between 2009 and 2020. Tinzaparin was delivered as a single-bolus injection at time 0 with dosing on the basis of doubling of standard 4-hour session dose. Tinzaparin efficacy was examined using visual observations (score 1–4) of the dialyzer and venous bubble trap at the end of dialysis and using anti-Xa measured at 15 and 30 minutes and 1, 2, 4, 6, and 8 hours after HD start.

**Results:**

Forty-seven patients were included. The mean tinzaparin dose was 107±20 IU/kg. Anti-Xa levels peaked at 15 minutes with 1.3±0.4 IU/ml and progressively declined reaching 0.9±0.3 IU/ml at 1 hour, 0.4±0.21 IU/ml at 4 hours, and 0.15±0.15 IU/ml at 8 hours. After the 8-hour session, none of the patients had severe clotting of their dialyzer or venous chamber. Moderate blood clotting was observed in the dialyzer of 6 patients (20%) and in the venous chamber of 22 patients (61%). On the basis of the simulation results, tinzaparin dose was increased in 27 patients (58%) with a mean home-discharge dose of 123±28 IU/kg.

**Conclusions:**

This study shows that anti-Xa levels stabilized rapidly after administration of tinzaparin for 8-hour HD. Administration of a single-bolus tinzaparin at the start of an 8-hour dialysis session seemed effective, although dose adjustment may be required.

## Graphical abstract

**Figure:**
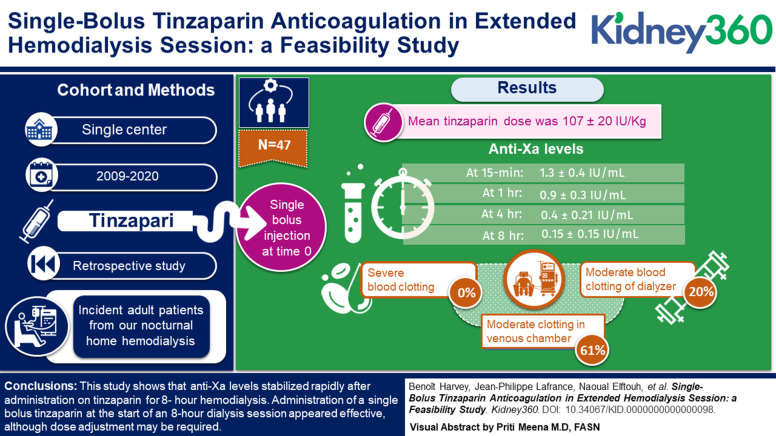

## Introduction

Anticoagulation is a key component of hemodialysis (HD) therapy to prevent clotting and deliver quality HD. Unfractionated heparin (UFH) has been used for decades during HD treatments and is still the most frequent anticoagulation strategy in many countries.^1^ Nonetheless, use of low-molecular weight heparin (LMWH) for extracorporeal circuit anticoagulation is common in many regions including Europe where its use was recommended in previous European Best Practices Guidelines as far as in 2002.^2,3^

Randomized and nonrandomized studies have shown comparable efficacy and safety of LMWH compared with UFH for conventional 4-hour dialysis sessions.^4–6^ Advantages of LMWH over UFH are mostly related to LMWH single-dose bolus injection and include ease of utilization, minimization of potential manipulation errors, and reduction in nursing-related tasks.^2,7,8^ Use of LMWH anticoagulation for HD has been associated with positive effect on lipid profile and possibly improved cost-effectiveness without increasing bleeding risk.^7–10^ Equipoise persist in regard to osteoporosis with studies showing similar^11,12^ or reduced^13^ risk with LMWH compared with UFH.

Among LMWH molecules, tinzaparin may be a first-choice molecule for HD anticoagulation because of its endothelial degradation, hence limiting potential accumulation with kidney failure.^14^ HD anticoagulation with tinzaparin has been evaluated in observational studies with positive outcomes.^7–9, 15^ However, very limited data are available for home and extended HD.^3,16^ Considering the increasing international use of home and intensive HD, evaluation of anticoagulation options in these patients is essential. The main objectives of this study were to evaluate the safety and efficacy of tinzaparin for anticoagulation of the extracorporeal circuit in extended, 8-hour, HD sessions.

## Methods

### Study Population and Design

This retrospective single-center cohort study included incident adult patients from our nocturnal home HD (HHD) program between 2009 and 2020. Patients who never received tinzaparin for conventional HD anticoagulation (routinely used in our center) and those without measurement of anti-Xa levels during their simulation session were excluded.

Patients referred to our nocturnal HD program had their first 8-hour HD session (simulation) in the hospital, at which time they received twice the tinzaparin (Innohep, Leo Pharmaceuticals, Canada) dose required for a standard 4-hour HD session. This initial 4-hour tinzaparin dose was derived from a rounded weight-based dose (60 IU/kg) with further adjustment (1000 IU increase or decrease) for bleeding or clotting risks in individual patients, on the basis of our in-center HD unit algorithm. Tinzaparin was administrated as a single-dose injection through the venous circuit line at time zero of the 8-hour HD session.

### Outcomes and Definitions

The study primary outcome was the efficacy of tinzaparin anticoagulation in extended HD. Efficacy was visually assessed by evaluation of clot formation of the dialyzer and the venous bubble trap using a four-point semiquantitative scoring system. This scoring system has been used and validated in previous HD studies and is defined as grade 1 (clear), grade 2 (presence of fibrin), grade 3 (presence of blood clots), and grade 4 (complete coagulation).^15,17^ For all patients, evaluation of the dialyzer and venous bubble air trap was performed at the end of the HD session by the senior HHD nurse, with a total of three different nurses involved over the study duration.

The secondary outcomes of this study included tinzaparin dose/response profile, which was assessed through measurement of anti-Xa levels at time 0 and after 15 and 30 minutes, and 1, 2, 4, 6, and 8 hours of HD treatment. We also evaluated predictors of anti-Xa levels at 15 minutes and explored characteristics associated with tinzaparin home-discharge dose.

With the exception of time 0 dosing, blood samplings were performed through the arterial circuit line with ongoing blood pumping. Anti-Xa levels were measured using an anti-Xa colorimetric assay (Stago-Liquid 00311US, Diagnostica Stago Inc), with anti-Xa considered the gold standard for monitoring LMWH activity.^18^ Concurrently, other coagulation markers were obtained, including prothrombin time (PT), international normalized ratio (INR), and activated cephalin time (ACT). In addition, blood pressure, hemoglobin, platelets count, weight, and ultrafiltrate volume were collected before and/or after the simulation session. All baseline characteristics, including race, were collected through electronic health record and defined at HHD training start. This study was approved by Hôpital Maisonneuve-Rosemont research ethic board (no. 20181228) and was conducted in accordance with the Declaration of Helsinki.

### Statistical Analysis

In all descriptive statistics, continuous data are presented as mean with SD for normally distributed variable and median with interquartile range for non-normally distributed variable. Categorical variables are displayed as count and percentage.

Predictors of anti-Xa levels 15 minutes after tinzaparin dose and the association between baseline characteristics and home-discharge tinzaparin dose were assessed in linear regressions. Variables were included in the multivariable regressions when their unadjusted *P*-value was <0.2, with the exception of age and sex that were prespecified in all models. A two-tailed *P*-value <0.05 was considered statistically significant. All statistical analyses were performed by N. Elftouh using SAS version 9.4 (SAS Institute, Cary, NC).

## Results

**Figure 1:**
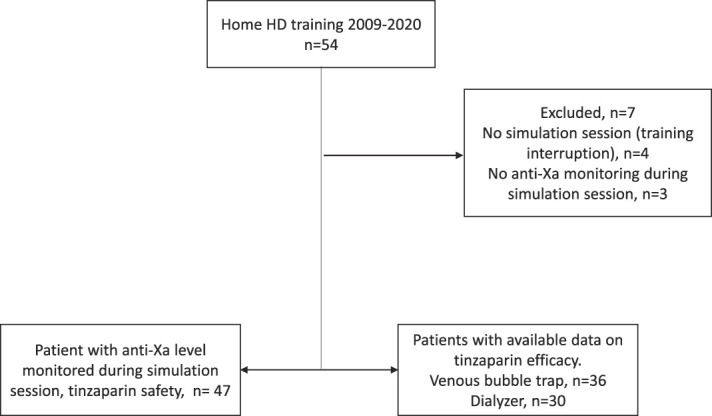
Study population.

Of the 54 patients who initiated nocturnal HHD in our program between 2009 and 2020, seven were excluded due to unavailability of anti-Xa measurement during their 8-hour simulation (*n*=3) or lack of simulation due to training interruption (*n*=4); hence, 47 patients were included in the study (Figure 1). Baseline characteristics of the cohort are presented in Table 1. The mean patient age was 45±14 years, with 72% men and 33% having diabetic kidney disease. The body mass index (BMI) was 29±7 kg/m^2^, and 25 patients (53%) were dialyzed using an arteriovenous fistula (AVF). Twenty-four patients (51%) were receiving acetylsalicylic acid (ASA), warfarin, and/or clopidogrel. All HD sessions were performed using a 2.2 m^2^ filter with a blood flow of 300 ml/min, dialyze flow of 350 ml/min, and using AK94, AK95, AK96, or AK98 (Baxter/Gambro) HD machines. The mean tinzaparin dose for the 8-hour session was 8772±2121 IU, representing a weight-based dose of 107±20 IU/kg (Table 2). Mean ultrafiltration (*n*=45) was 2.5±1.4 L during the simulation session.

**Table 1:** Baseline characteristics of the study population

Demographics	Count (%) *n*=47
Age (yr)	45.4±14.1
Female	13 (28)
BMI (kg/m^2^), *n*=41	28.7±7.2
**Race**	
Black	5 (11)
White	37 (79)
Other	5 (11)
Smoking, past or current	21 (45)
**Causes of kidney disease**	
Diabetes mellitus	15 (33)
Hypertension	6 (13)
Glomerulonephritis	14 (30)
Other	10 (22)
Coronary artery disease	9 (19)
Peripheral vascular disease	5 (11)
Diabetes mellitus	20 (43)
Chronic obstructive pulmonary disease	6 (13)
Cerebrovascular disease	3 (6)
Previous or active cancer	7 (15)
**Anticoagulation or antiplatelets**	
ASA	18 (38)
Clopidogrel	2 (4)
Warfarin	4 (9)
Predialysis follow-up	25 (53)
**Vascular access**	
AVF	25 (53)
CVC	22 (47)
Systolic blood pressure (mm Hg), *n*=37	146±26
Diastolic blood pressure (mm Hg), *n*=37	89±16
Hemoglobin (g/dl), *n*=40	114±15
Platelets (10^9^/L), *n*=39	204±61
Weight (kg), *n*=45	83.1±22.8

Data are presented as mean±SD or number (%). Data available for 47 patients, except if specified otherwise. BMI, body mass index; ASA, acetylsalicylic acid; AVF, arteriovenous fistula; CVC, central venous catheter.

**Table 2:** Tinzaparin dose during the 8-hour simulation session and home-discharge dose

Tinzaparin Dose	Mean Dose (SD)	Minimum	Maximum
Simulation dose (IU)	8772 (2121)	5000	14,500
Simulation dose (IU/kg)	107 (20)	68	152
Home-discharge dose (IU)	9171 (2351)	5000	14,500
Home-discharge dose (IU/kg)	120 (26)	77	196

### Tinzaparin Efficacy

Measures of tinzaparin efficacy during the 8-hour HD session were based on a semiquantitative assessment and are presented in Table 3. Dialyzer clotting was grade 1 (clear) for 14 patients (47%), grade 2 (presence of fibrin) for ten patients (33%), and grade 3 (presence of blood clots) for six patients (20%). None of them had a grade 4 clotting although data were unavailable for 17 patients. Thirty-six patients were evaluated for venous air trap clotting degree with six patients (17%) categorized as grade 1, eight patients (22%) classified as grade 2, 22 patients (61%) identified as grade 3, and none with grade 4.

**Table 3:** Evaluation and appearance of dialyzer and bubble trap

Clotting Grade	Count (%)
**Dialyzer clotting (*n*=30)**	
Stage 1 (clear)	14 (47)
Stage 2 (presence of fibrin)	10 (33)
Stage 3 (presence of blood clots)	6 (20)
Stage 4 (complete coagulation of the dialyzer)	0
**Venous trap clotting (*n*=36)**	
Stage 1 (clear)	6 (17)
Stage 2 (presence of fibrin)	8 (22)
Stage 3 (presence of blood clots)	22 (61)
Stage 4 (complete occlusion of air trap)	0

**Figure 2:**
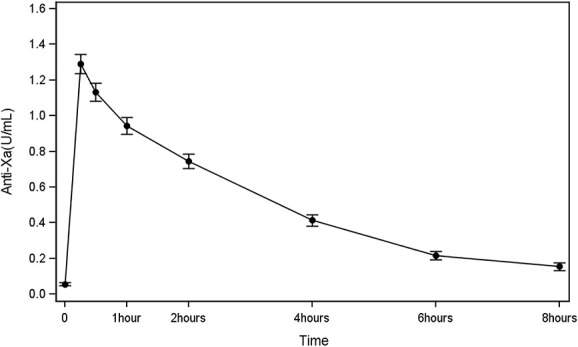
**Mean measurements of anti-Xa levels (mean±SD) for each time point during the 8-hour HD session.** Anti-Xa levels 1.29±0.36 IU/ml at 15 minutes, 1.13±0.33 IU/ml at 30 minutes, 0.94±0.30 at 1 hour, 0.74±0.277 at 2 hours, 0.41±0.21 at 4 hours, 0.22±0.26 at 6 hours, and 0.15±0.15 at 8 hours.

**Figure 3:**
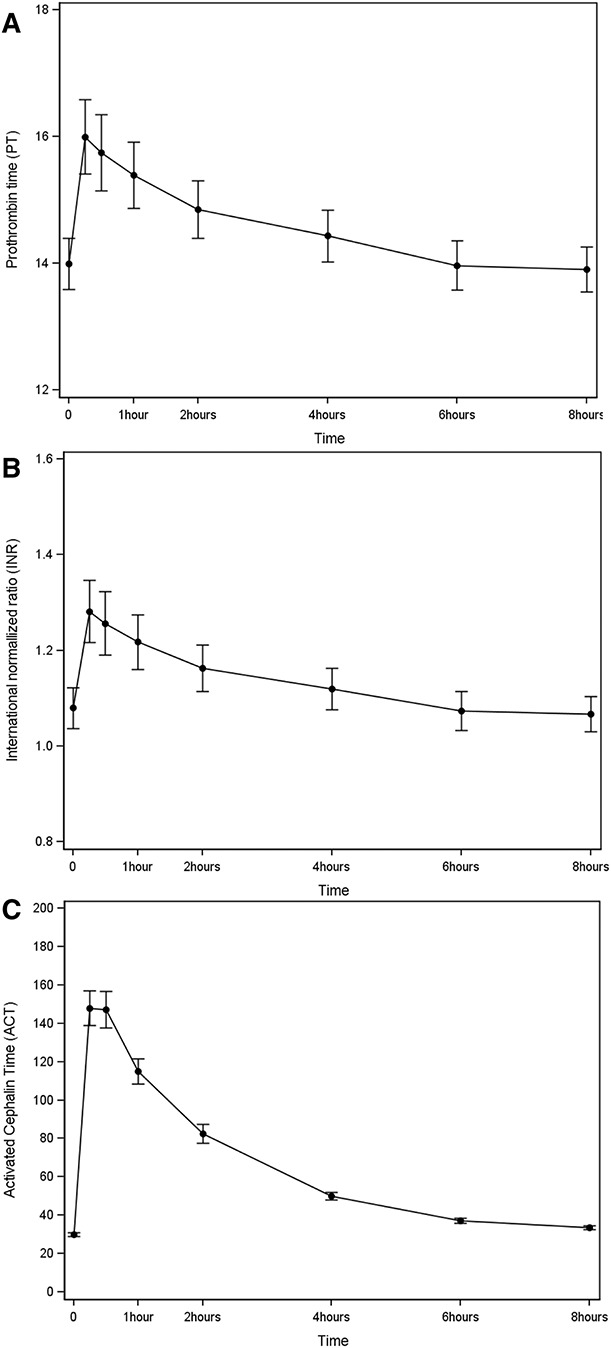
Mean measurements of coagulation markers during the 8-hour hemodialysis session. A) INR, B) ACT, and C) PT.

Mean measurements of anti-Xa levels at each time point during the 8-hour session are shown in Figure 2 and Supplemental Table 1. Anti-Xa levels peaked 15 minutes after tinzaparin administration with a mean level of 1.29±0.36 IU/ml and then progressively declined at 0.94±0.30 IU/ml after 1 hour, 0.41±0.21 IU/ml after 4 hours, 0.22±0.16 IU/ml after 6 hours, and 0.15±0.15 IU/ml after 8 hours. A similar relationship was seen with PT, INR, and ACT, as shown in Figure 3. None of the patients had bleeding events during their 8-hour session. Distribution of anti-Xa levels at 8 hours, stratified by clotting grade is shown in Supplemental Figure 1.

After the simulation session, tinzaparin doses adjustment were made based on visual clotting scores assessed at the end of the 8-hour session and anti-Xa levels. Changes were based on clinical judgment and the availability of tinzaparin prefilled syringes combinations.^19^ The mean home-discharge dose was 123±28 IU/kg, ranging from 77 to 196 IU/kg. Specifically, 17 patients (38%) stayed on the same dose as their simulation dose, 27 (58%) required a higher dose, and 2 patients (4%) required a lower dose (Table 2).

### Predictors of Anti-Xa Level and Tinzaparin Dose

In a multivariable linear regression, BMI (*B* 0.02 per unit increase, 95% confidence interval [CI], 0.00 to 0.04, *P*=0.02), hemoglobin level (*B* 0.02 per 1 g/L increase, 95% CI, 0.01 to 0.02, *P*<0.001), and use of a central venous catheter (CVC) (compared with an AVF; *B* 0.28, 95% CI, 0.08 to 0.47, *P*<0.007) were independently associated with higher anti-Xa peak levels at 15 minutes. There was also a trend toward higher anti-Xa level among women compared with men (*B* 0.17, 95% CI, −0.02 to 0.36, *P*=0.08). Age was not associated with anti-Xa level (Table 4). Several characteristics were associated with the home-discharged tinzaparin dose (in IU/kg) although none remained statistically significant after adjustment, although higher BMI was associated with a trend toward a lower home-discharged dose (*B* −1.36 per kg/m^2^, 95% CI, −2.9 4 to 0.23, *P*=0.09) (Table 5).

**Table 4:** Adjusted predictors of anti-Xa levels 15 minutes after tinzaparin administration

Variables	Coefficient	95% CI	*P* Value
Intercept	1.15	0.99 to 1.31	<0.001
Female (versus male)	0.17	−0.02 to 0.36	0.09
Age, per yr	−0.006	−0.014 to 0.002	0.16
BMI, per kg/m^2^	0.02	0.00 to 0.04	0.02
Hemoglobin, per g/L	0.02	0.01 to 0.02	<0.001
CVC (versus AVF)	0.28	0.08 to 0.47	0.007

CI, confidence interval; BMI, body mass index; CVC, central venous catheter; AVF, arteriovenous fistula.

**Table 5:** Adjusted predictors of home-discharge tinzaparin dose

Variables	Coefficient	95% CI	*P* Value
Intercept	124.8	109.1 to 140.5	<0.001
Female (versus male)	−4.9	−23.0 to 13.1	0.58
Age, per yr	−0.11	−0.94 to 0.71	0.78
BMI, per kg/m^2^	−1.36	−2.94 to 0.23	0.09
Hemoglobin, per g/L	0.27	−0.36 to 0.90	0.39
CVC (versus AVF)	5.9	−11.6 to 23.4	0.49
Anticoagulation or antiplatelet use	−9.9	−32.5 to 12.51	0.37

CI, confidence interval; BMI, body mass index; CVC, central venous catheter; AVF, arteriovenous fistula.

## Discussion

In this feasibility study, we assessed the efficacy of a single bolus of tinzaparin during 8-hour extended HD sessions. Overall, tinzaparin proved to be effective. There were no reports of severe clotting in the dialyzer and venous chamber at the end of the 8-hour session and no acute bleeding event. However, among the patients whose visual clotting assessment data were available, moderate blood clotting was observed in the dialyzer of 6 patients (20%) and in the venous chamber of 22 patients (61%). Consequently, tinzaparin dose was increased in 58% of patients, with a mean simulation dose of 107±20 IU/kg and mean home-discharge dose of 120±26 IU/kg.

Anti-Xa level is a reliable surrogate marker to illustrate the degree of activity and assess the risk of bioaccumulation of LMWH, including tinzaparin.^19,20^ Although there are no guidelines for a specific range of therapeutic anti-Xa levels for anticoagulation during HD, official drug monograph information suggests a lower threshold of 0.2 anti-Xa IU/mL and an upper threshold around 0.4 IU/mL.^19,20^ For references, anti-Xa targets for therapeutic anticoagulation, such a venous thromboembolism, may reach 1.0 IU/mL.^21^ In this study, we observed that tinzaparin activity peaked after 15 minutes with a mean anti-Xa level of 1.29±0.36 IU/ml and then rapidly stabilized, as reported previously in 4-hour HD.^22^ Over the course of the 8-hour HD session, anti-Xa levels reached near subtherapeutic levels after 6 hours (0.22±0.16 IU/ml), thus potentially increasing the risk of clotting in the dialyzer/venous air trap for the past 2 hours of HD.

To the best of our knowledge, only one study previously assessed the efficacy of tinzaparin during extended HD. Bugeja *et al.* evaluated tinzaparin use in 16 patients receiving nocturnal HD during a total of 177 in-center 6–8 hour HD sessions.^23^ Their dosing regimen consisted of two tinzaparin boluses given at the start of dialysis and mid-dialysis with each bolus representing 25% of the UFH dose used for each patient. Overall, 5 patients (31%) required an increase in dosage, and no bleeding events were reported.^23^ Their study was not powered to assess predictors of anti-Xa levels.

Another study by Verhave *et al.* compared two different LMWH drugs (dalteparin and nadroparin), using two-dose regimens with both medication.^24^ In their study, nadroparin was associated with prolonged anti-Xa activity compared with dalteparin. These two studies used a two-dose regimen, with administration of the second LMWH dose at midsession, usually at the 4-hour mark. A midsession LMWH administration is poorly compatible with use of nocturnal HHD because patients are usually sleeping at the time when this second dose is needed. In this regard, the single-bolus tinzaparin administration used in our study is much more convenient for patients performing nocturnal home treatments.

A previous study by Huang *et al.* assessed a single dose of dalteparin in eight patients on nocturnal HD using predetermined dose adjustments. Of these, five patients had to switch back to UFH within 4 weeks because of high clotting scores at the maximum predetermined dalteparin dose.^25^ This demonstrates the importance of individualized dose adjustments based not only on weight but also on the patient's response.

Our study was not designed to compare LMWH and UFH, although previous studies and trials have overall shown similar efficacy of LMWH when compared with UFH for 4-hour HD sessions.^4,5^ LMWH offers practical advantage and may be associated with lower bleeding risk and favorable effect on lipid profile.^4–6^ Extended HD sessions are probably prone to the same advantages than standard 4-hour sessions regarding single-dose LMWH administration. Moreover, patients performing HHD might gain further benefits from the single-dose administration of LMWH compared with the bolus and continuous perfusion required with UFH anticoagulation, limiting the risk of errors with different doses of UFH syringes.

This study is among the firsts to determine predictors of peak anti-Xa levels at 15 minutes with a LMWH in a HD setting, where BMI, hemoglobin, and use of CVC (compared with an AVF) were independently associated with higher peak anti-Xa levels. Use of CVC was also associated with higher dalteparin doses in a study with pediatric HHD patients, likely because of higher blood flow rates with an AVF than a CVC.^26^ The association between higher BMI and increased anti-Xa levels is likely related to initial weight-based tinzaparin prescription, although increased risk of supratherapeutic anticoagulation have previously been raised for obese patients receiving LMWH.^19,20^ Of notes, our mean anti-Xa level at 15 minutes was similar than what was previously reported for patients pursuing 4-hour session.^22^

This study has several strengths. It is the first detailed report of a single tinzaparin dose in extended HD. Compared with most studies assessing LMWH in HD, evaluation of anti-Xa levels was performed at more frequent intervals, including only 15 minutes after the dose administration leading to the detection of a more optimal peak dose compared with when anti-Xa value is first assessed after 1 hour post-LMWH administration. By including early and frequent anti-Xa level monitoring throughout the dialysis session, we were able to evaluate the efficacy of the single-dose LMWH approach more thoroughly than what was previously performed. Our study sample size was also larger than in other similar studies. These strengths need to be balanced against the study limitations. The single-center design limits generalization of the study results. There was a significant amount of missing data because of the retrospective nature of the study. We also did not have details on patient-specific conventional HD (4-hour) tinzaparin dose used before the simulation sessions. Importantly, our study did not assess long-term use nor safety (including bleeding events) of LMWH during extended HD session because the study design was based on a single 8-hour session.

In conclusion, this study demonstrates the safety and efficacy of a single-bolus dose of tinzaparin in extended 8-hour HD. Doubling the standard 4-hour dose as an initial dosing regimen seemed to be a reasonable approach, although additional dose adjustments might be required. Further studies should explore long-term outcomes of tinzaparin use in nocturnal HHD.

## Supplementary Material

SUPPLEMENTARY MATERIAL

## Data Availability

Data cannot be shared: Current Research Ethic Board approval does not support data sharing.

## Supplemental Material

Supplemental Table 1. Levels of anti-Xa, PT, TCA, and INR at different time points, including number of included and missing samples.

Supplemental Figure 1. Anti-Xa level at the end of the 8-hour HD session, by (A) venous trap and (B) dialyzer clotting grade.
