# Supplementary material for: Single-Bolus Tinzaparin Anticoagulation in Extended Hemodialysis Session: A Feasibility Study
Source: Kidney360. 2023 Mar 15;4(5):641–7. doi: 10.34067/KID.0000000000000098 (PMC10278850; doi:10.34067/KID.0000000000000098)

Supplements

Table S1. Levels of anti-Xa, PT, TCA and INR at different timepoints, including number of included and missing samples.

	**Number**	**Missing value**	**Levels (mean ± standard deviation)**
**Anti-Xa**			
15-minute	44	3	1.29 ± 0.36
30-minute	43	4	1.13 ± 0.33
1-hour	42	5	0.94 ± 0.30
2-hour	46	1	0.74 ± 0.27
4-hour	44	3	0.41 ± 0.21
6-hour	45	2	0.22 ± 0.16
8-hour	45	2	0.15 ± 0.15
**PT**			
15-minute	43	4	15.99 ± 2.65
30-minute	43	4	15.74 ± 3.85
1-hour	42	5	15.39 ± 3.39
2-hour	45	2	14.85 ± 3.05
4-hour	44	3	14.43 ± 2.69
6-hour	44	3	13.97 ± 2.60
8-hour	46	1	13.90 ± 2.41
**TCA**			
15-minute	39	8	147.88 ± 56.82
30-minute	42	5	147.16 ± 61.87
1-hour	41	6	114.94 ± 41.95
2-hour	45	2	82.47 ± 33.14
4-hour	44	3	49.90 ± 13.79
6-hour	44	3	37.09 ± 8.39
8-hour	46	1	33.41 ± 6.60
**INR**			
15-minute	43	4	1.08 ± 0.28
30-minute	43	4	1.28 ± 0.42
1-hour	42	5	1.22 ± 0.37
2-hour	45	2	1.16 ± 0.33
4-hour	44	3	1.12 ± 0.28
6-hour	44	3	1.07 ± 0.27
8-hour	46	1	1.07 ± 0.25

Figure S1 Anti-Xa level at the end of the 8-hour HD session, by (A) venous trap and (B) dialyzer clotting grade.

A)

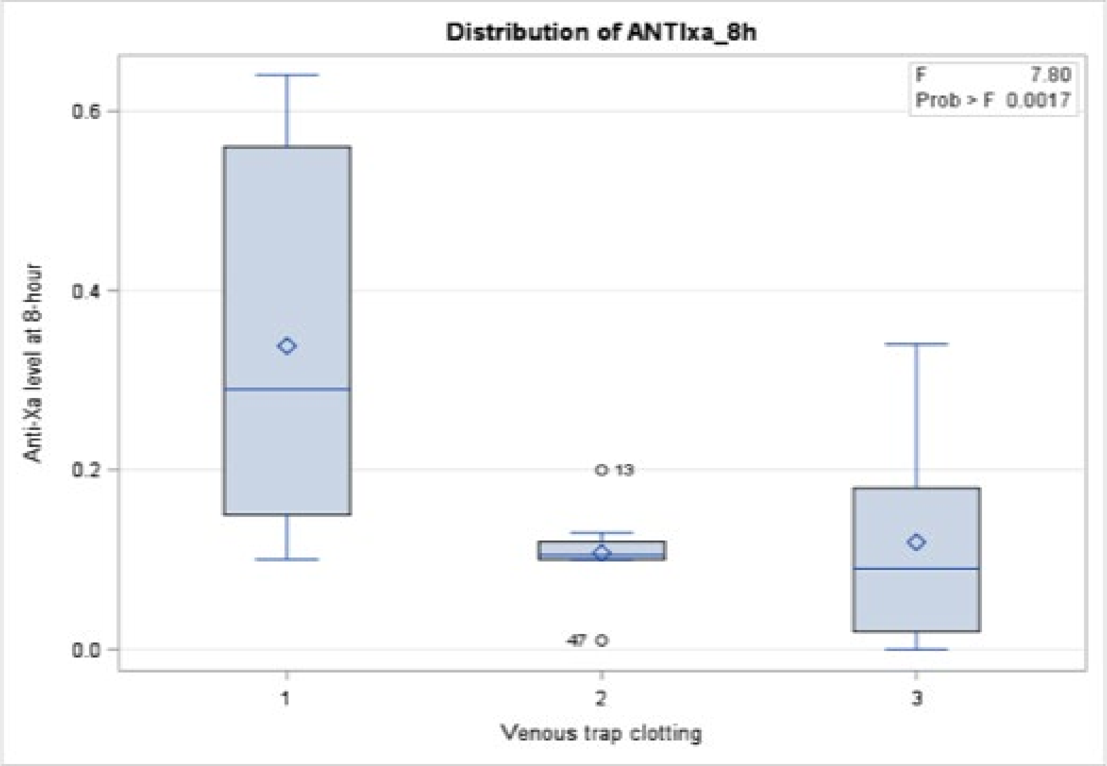

B)

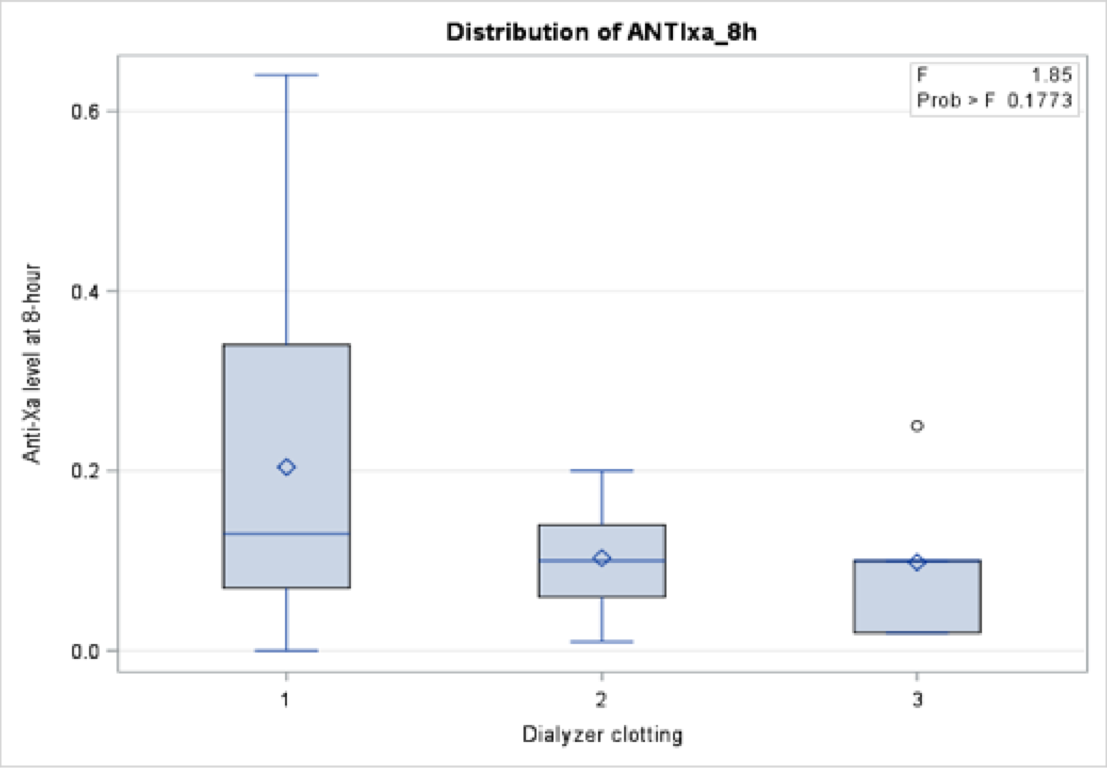

Supplement: SUPPLEMENTARY MATERIAL [file kidney360-4-641-s001.pdf]
